# Prevalence, vector evolution, and visual impact of astigmatism: a large-scale study of 79,066 children in Beijing, China

**DOI:** 10.3389/fmed.2026.1837925

**Published:** 2026-05-11

**Authors:** Jingjing Wang, Yunsheng Zhang, Hui Wang, Xiaolan Xie, Bidan Zhu

**Affiliations:** 1 Department of Ophthalmology, Tongzhou Maternal and Child Health Hospital of Beijing, Beijing, China; 2 Department of Neurosurgery, China-Japan Friendship Hospital, Beijing, China; 3 Department of Neurosurgery, Civil Aviation General Hospital, Beijing, China; 4 Department of Ophthalmology, Beijing Chaoyang Hospital, Capital Medical University, Beijing, China

**Keywords:** astigmatism, epidemiology, myopia progression, uncorrected visual acuity, with-the-rule

## Abstract

**Background:**

Astigmatism is highly prevalent among school-aged children. This study aimed to evaluate the epidemiological distribution of astigmatism in a large-scale Chinese pediatric population and investigate the independent and interactive impacts of astigmatism magnitude and axis on uncorrected visual acuity (UCVA).

**Methods:**

This school-based, cross-sectional study conducted in Tongzhou District, Beijing, included 79,066 students aged 6 to 20 years (mean age: 11.35 ± 3.23 years). Participants underwent standardized non-cycloplegic autorefraction and UCVA assessment. Astigmatism was quantified by cylinder power and decomposed into Thibos vector components (*J*_0_, *J*_45_). Generalized linear models (GLMs) were utilized to determine the independent association between astigmatism axis and visual function, adjusting for spherical power and astigmatism magnitude.

**Results:**

The overall prevalence of astigmatism was 43.6%, pre-dominantly characterized by mild severity and the with-the-rule (WTR) subtype (82.9%). Both prevalence and severity increased progressively with advancing educational stages. Crucially, multivariate analysis revealed that after adjusting for spherical equivalent (*SE*), advancing grade was independently associated with a slight against-the-rule (ATR) and oblique shift. Furthermore, males exhibited a higher susceptibility to the WTR orientation, whereas females were more prone to ATR and oblique. Regarding visual performance, astigmatism magnitude significantly degraded UCVA in non-myopic and low-myopic eyes, but this effect was saturated and masked in moderate-to-high myopia. Although the WTR axis was associated with a statistically worse UCVA compared to oblique subtypes, the absolute difference was clinically negligible, likely reflecting statistical amplification within this mega-cohort.

**Conclusion:**

WTR is the pre-dominant astigmatism subtype in school-aged children, propelled by the concurrent myopization process. While the magnitude of astigmatism profoundly impairs UCVA—heavily modulated by the concurrent *SE*—the functional impact of axis orientation remains clinically insignificant. These findings suggest that targeted monitoring and correction strategies may benefit from being tailored to specific genders and refractive profiles.

## Introduction

Astigmatism is a prevalent refractive error characterized by the inability of the eye to focus light uniformly on the retina, resulting in blurred or distorted vision at all distances. Significant uncorrected astigmatism is not merely a static optical defect; it is a dynamic component of refractive development that has been implicated in the acceleration of myopic progression (1) and an elevated risk of meridional amblyopia (2–4). Epidemiological studies indicate that the prevalence of astigmatism in school-aged children varies widely, with reported rates ranging from 10% to over 70% across different populations (4–7). Therefore, characterizing the distribution and functional impact of pediatric astigmatism is crucial for comprehensive vision care.

Traditionally, astigmatism is classified based on the orientation of the steepest meridian into with-the-rule (WTR), against-the-rule (ATR), and oblique subtypes. While the general epidemiology of pediatric astigmatism is well-documented, the dynamic longitudinal trajectory of its axis remains poorly understood. Specifically, how the astigmatic axis structurally evolves across different educational stages and biologically interacts with the concurrent myopization process (axial elongation) in school-aged populations is a critical piece of the refractive puzzle that is often overlooked in traditional magnitude-centric analyses.

Furthermore, existing literature has pre-dominantly focused on the prevalence and magnitude of astigmatism, leaving a critical gap regarding the functional impact of its axis (5, 6). While the magnitude of astigmatism is a well-established driver of visual impairment, the specific impact of axis orientation on uncorrected visual acuity (UCVA) remains a subject of ongoing debate (8, 9). In particular, there is remarkably limited large-scale data rigorously analyzing the independent contribution of axis orientation to UCVA after controlling for the confounding effect of spherical power in a myopia-predominant cohort.

To address these gaps, we conducted a large-scale cross-sectional study involving nearly 80,000 students in Beijing. By utilizing both conventional categorical notation and continuous vector analysis (J_0_ and J_45_), we aimed to: (1) evaluate the grade-dependent evolution of astigmatism prevalence and severity; (2) disentangle the complex interplay between astigmatism axis, advancing educational stages, and myopic shift; and (3) rigorously investigate the independent and interactive effects of astigmatism magnitude and axis on UCVA.

## Methods

### Study design and participants

This large-scale, school-based cross-sectional study was conducted in Tongzhou District, Beijing, between April and May 2025. As the designated municipal sub-center of Beijing, Tongzhou serves as a representative region reflecting the characteristics of super cities in northern China. The protocol adhered to the Declaration of Helsinki with ethical approval and informed consent obtained from the students' parents or legal guardians.

To minimize selection bias, a comprehensive, school-based enumeration approach was adopted. All students enrolled in the 71 participating primary and secondary schools were invited to participate in the screening program. From the initial 81,816 students screened, the explicit inclusion criteria were: those who provided written informed consent. To prevent confounding effects on natural refractive status and visual acuity, the exclusion criteria were strictly defined as follows: (1) current or recent (within the past 3 months) use of orthokeratology (OK) lenses, rigid gas permeable (RGP) lenses, or atropine eye drops; (2) history of ocular trauma or refractive surgery; (3) presence of organic ocular pathologies affecting vision (e.g., manifest strabismus, amblyopia, congenital cataracts, glaucoma, or severe ptosis); (4) inability to cooperate with the examinations; and (5) missing or incomplete core data. Ultimately, 79,066 students (mean age: 11.35 ± 3.23 years) were included in the final analysis after data cleaning.

### Ophthalmic examinations

Examinations were conducted by trained technicians under a standardized protocol. UCVA was measured at a standardized distance of 5 meters under controlled ambient classroom lighting (approximately 300–500 lux) using a retro-illuminated logMAR chart with tumbling-E optotypes (Standard Chinese Logarithmic Visual Acuity Chart, GB 11533-2011). Non-cycloplegic autorefraction was performed using a desktop autorefractor (Topcon RM-800, Topcon Corp., Tokyo, Japan) equipped with a standard auto-fogging mechanism to relax accommodation. To further minimize accommodative bias, all examinations were strictly conducted in a standardized environment with controlled lighting to reduce proximal stimuli. Technicians ensured proper head positioning and instructed students to maintain steady fixation on the internal target. The average of three consecutive measurements was recorded. If the spherical power difference between readings exceeded 0.50 D, measurements were repeated until consistency was achieved. For quality control, 5% of students were randomly re-examined.

### Definitions and classifications

Refractive error was recorded in sphere (S), cylinder (C), and axis (A). The spherical equivalent (*SE*) was calculated as *SE* = S + 1/2C. Myopia was defined as *SE* ≤ −0.50 D, hyperopia as *SE* ≥ +0.50 D, and astigmatism as absolute cylinder power (|C|) ≥ 0.75 D. Astigmatism was reported in negative cylinder form and classified by severity: mild (0.75 D ≤ |C| ≤ 1.5 D), moderate (1.5 D < |C| ≤ 3.0 D), and severe (|C| > 3.00 D). Astigmatism axis was classified according to standards: WTR as axis 180 ° ± 30 °, ATR as axis 90 ° ± 30 °, and oblique astigmatism as the remaining axes.

### Data management and statistical analysis

Data were collected using a digital system with unique QR codes and uploaded to a centralized platform audited for accuracy. Given the high inter-ocular correlation of astigmatism parameters (Pearson's correlation coefficient *r* = 0.760, *P* < 0.001), only right eye data were analyzed for prevalence and risk factors. However, absolute values of cylinder and *SE* were used to assess the impact on visual acuity.

An *a priori* sample size calculation was performed using $N=\frac{Z^{2}\times P\left(1-P\right)}{d^{2}}\;$to ensure adequate power. Assuming a conservative 50% astigmatism prevalence (10) to maximize the requirement, a 95% confidence level (*Z* = 1.96), and a 1% margin of error (*d*), the minimum sample size was 9,604. Our final cohort of 79,066 vastly exceeded this, providing >99% statistical power for all analyses.

All statistical analyses were performed using SPSS (version 27.0; IBM Corp., Armonk, NY, USA) R software (version 4.2.2, R Foundation for Statistical Computing). Prior to analysis, the assumptions for parametric testing were rigorously verified. Normality was initially assessed using the Kolmogorov-Smirnov test. Although formal normality was rejected—a common statistical artifact in exceptionally large sample sizes (*N* = 79,066) due to hyper-sensitivity to minor deviations—parametric tests were deemed robust and appropriate based on the Central Limit Theorem. Homogeneity of variances was confirmed using Levene's test prior to variance analyses. For all multivariable regression models, multicollinearity was assessed using the Variance Inflation Factor (VIF), with all models yielding VIF values strictly < 5.0, indicating no significant collinearity among independent variables. Continuous variables were presented as mean ± SD or median (IQR: Q1–Q3), and categorical variables as frequencies (percentages) with 95% confidence intervals.

Pearson's χ^2^ test compared categorical distributions, while the linear-by-linear association test evaluated grade- and age-dependent trends in astigmatism severity and axis. One-way analysis of variance (ANOVA) assessed the linear trend of the continuous vector component *J*_0_ (*J*_0_ = (–C/2) cos(2α) across grade levels.

To identify independent determinants of astigmatic orientation, a multinomial logistic regression model (reference: WTR) was utilized because the dependent variable comprises three nominal, non-ordered categories. Furthermore, stratified multiple linear regression evaluated the specific association between astigmatism magnitude and UCVA across different myopia levels. A General Linear Model (GLM) was employed to compare LogMAR UCVA among axis subtypes because it effectively accommodates the simultaneous control of continuous covariates, including astigmatism magnitude, spherical power, and age. *Post-hoc* multiple comparisons were adjusted using the Bonferroni method. A two-tailed *P* < 0.05 was considered statistically significant.

## Results

### Demographic characteristics

As shown in Table 1, the school-based survey was conducted in the spring semester of 2025, including 79,066 primary and secondary school ranged from grade 1 to grade 12 students aged 6–20 years, of whom 40,286 were boys (51.0%) and 38,780 were girls (49.0%). The mean age of this cohort was 11.35 ± 3.23 years. The median cylinder power was 0.50 D (IQR: 0.25, 1.00 D, range: 0.00–6.00 D).

**Table 1:** Demographic and astigmatism prevalence of the surveyed students.

Feature	*N* (%)	Prevalence (%)
Age		
6	1,011 (1.3)	27.5
7	8,558 (10.8)	27.0
8	10,515 (13.3)	28.6
9	7,364 (9.3)	33.5
10	7,998 (10.1)	37.3
11	7,708 (9.7)	43.5
12	7,223 (9.1)	48.2
13	7,534 (9.5)	52.6
14	5,796 (7.3)	57.6
15	4,883 (6.2)	59.9
16	3,877 (4.9)	61.6
17	3,934 (5)	60.6
18	2,554 (3.2)	59.6
19	104 (0.1)	66.3
20	7 (0)	28.6
Educational stage		
Primary	49,250 (62.3)	35.1
Middle	18,911 (23.9)	55.8
Senior	10,905 (13.8)	60.9
Sex		
Boys	40,286 (51.0)	44.9
Girls	38,780 (49.0)	42.2
Astigmatism severity		
Without	44,597 (56.4)	56.4
Mild	23,838 (30.1)	30.1
Moderate	8,830 (11.2)	11.2
Severe	1,801 (2.3)	2.3
Astigmatism axis		
WTR	65,535 (82.9)	82.9
ATR	7,077 (9.0)	9.0
Obique	6,454 (8.2)	8.2
Total	79,066 (100.0)	100.0

Without: cylinder power less than 0.75 D.

WTR, with-the-rule; ATR, against-the-rule;

Percentages may not total 100.0 due to rounding.

### Overall prevalence and gender disparity

The overall prevalence of astigmatism (CYL ≥ 0.75 D) in this study was 43.6%. As expected mathematically, the prevalence demonstrated a strict downward trajectory as the definition became more stringent, dropping from 43.6% for astigmatism ≥ 0.75 D to 2.3% for significant astigmatism ≥ 3.00 D (Table 2).

**Table 2:** Prevalence of astigmatism at different thresholds and gender differences (%).

Prevalence (95% CI)	Astigmatism threshold			
	≥**0.75 D**	≥**1.50 D**	≥**2.00 D**	≥**3.00 D**
Overall	43.6 (43.2–43.9)	13.4 (13.2–13.7)	6.9 (6.7–7.1)	2.3 (2.2–2.4)
Boys	44.9 (44.4–45.4)	14.5 (14.2–14.8)	7.7 (7.5–8.0)	2.6 (2.5–2.8)
Girls	42.2 (41.7–42.7)	12.3 (12.0–12.7)	6.0 (5.8–6.2)	1.9 (1.8–2.1)
*OR*	1.12 (1.09–1.15)	1.20 (1.16–1.25)	1.31 (1.24–1.39)	1.37 (1.24–1.50)
χ^2^	59.844	78.641	93.034	42.268
*P*-value	< 0.001	< 0.001	< 0.001	< 0.001

OR, odds ratio.

Gender stratification revealed that boys consistently exhibited a significantly higher prevalence of astigmatism compared to girls across all severity thresholds (*P* < 0.001 for all). Specifically, at the overall ≥ 0.75 D threshold, this gender disparity was highly significant statistically (χ^2^ = 59.844, *P* < 0.001, Cramer's *V* = 0.028, indicating a small effect size). Notably, the gender disparity became more pronounced with increasing severity (*P*_*trend*_ < 0.001).

### Grade-dependent trends in astigmatism severity

The overall prevalence of astigmatism was 35.1% in primary school (grade 1–6), increased to 55.8% in junior high school (grade 7–9), and 60.9% in senior high school (grade 10–12). Statistically significant differences were observed among the three educational stages (χ^2^ = 3,925.092, *P* < 0.001, Cramer's *V* = 0.223), Table 3.

**Table 3:** Distribution of astigmatism severity across different educational stages. *N* (%).

Astigmatism severity	Primary	Junior	Senior	χ^2^	*P-value*	*P_*trend*_*
Without	31,974 (64.9)^a^	8,357 (44.2)^b^	4,266 (39.1)^c^	5,212.36	< 0.001	< 0.001
Mild	13,347 (27.1)^a^	6,724 (35.6)^b^	3,767 (34.5)^b^			
Moderate	3,273 (6.6)^a^	3,188 (16.9)^b^	2,369 (21.7)^c^			
Severe	656 (1.3)^a^	642 (3.4)^b^	503 (4.6)^c^			
Total	17,276 (35.1)	10,554 (55.8)	6,639 (60.9)	3,925.092	< 0.001	< 0.001

Total: includes the mild, moderate, and severe groups only.

P_trend_ indicates the P-value for the linear-by-linear association test.

Values sharing the same superscript letter (a, b, c) in the same row are not significantly different (P > 0.05).

**Figure 1:**
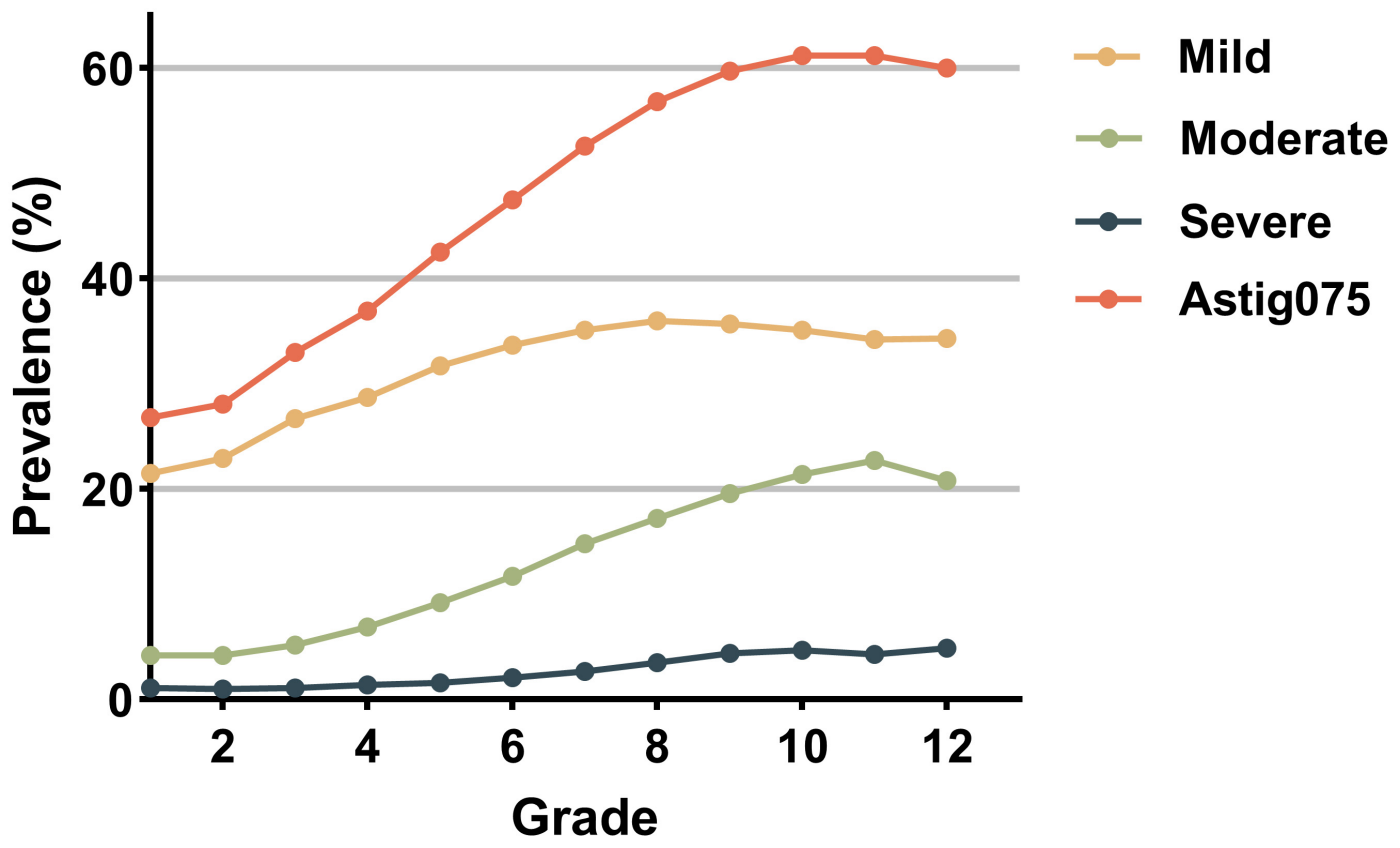
Grade-specific prevalence of astigmatism stratified by severity. The curves illustrate the prevalence trends of different astigmatism categories among students grade 1 to 12 years. The Astig075 represents the overall prevalence of clinically significant astigmatism (≥ 0.75 D), which serves as the sum of the Mild (0.75–1.50 D), Moderate (1.50–3.0 D), and Severe groups (CYL > 3.0 D).

**Figure 2:**
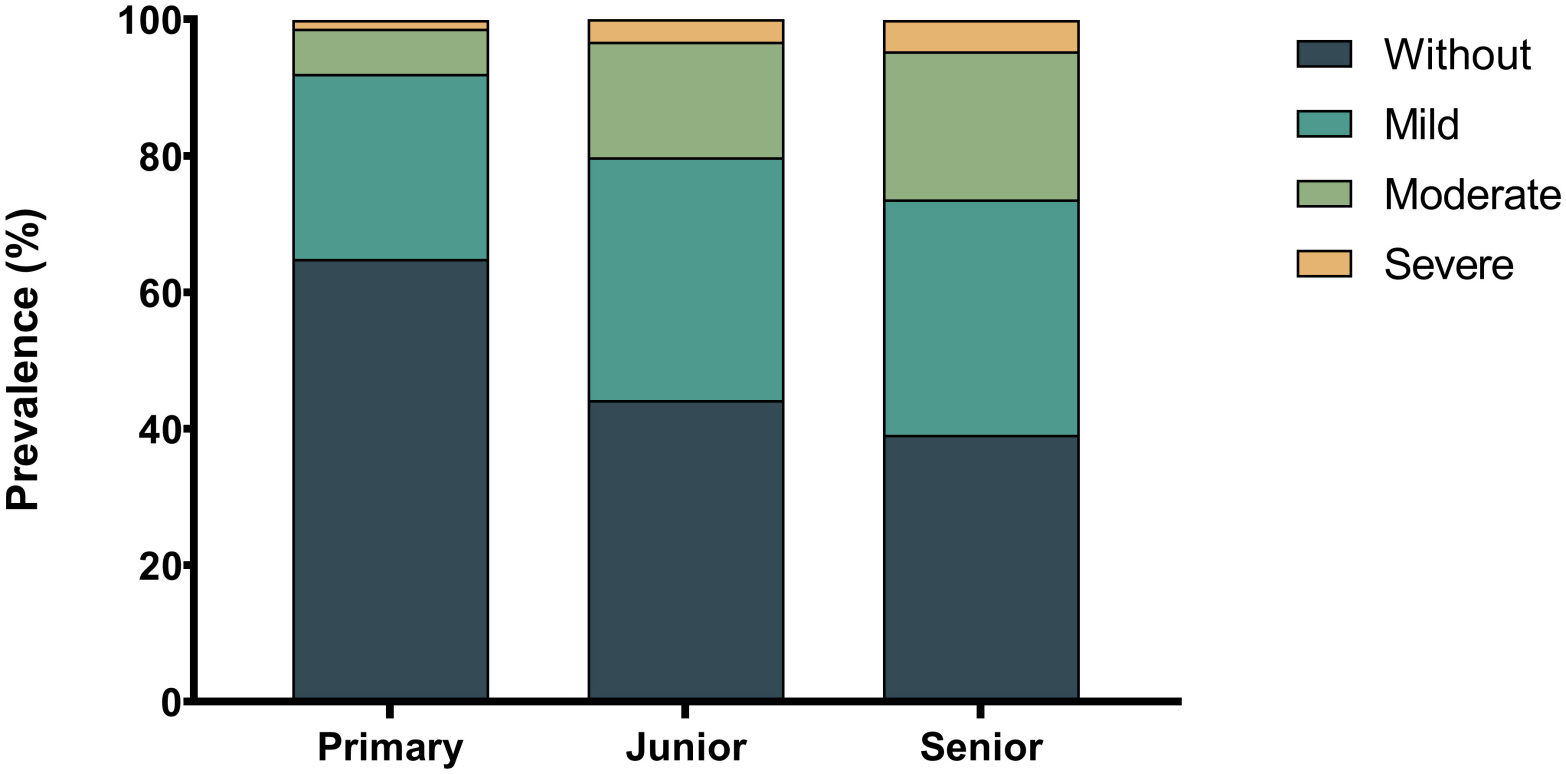
Prevalence and composition of astigmatism severity stratified by educational stage. The stacked bar chart illustrates the changing distribution of refractive status from Primary School to Senior High School. (Without: CYL < 0.75 D; Mild: 0.75–1.50 D; Moderate: 1.50–3.0 D; Severe: CYL > 3.0 D).

Longitudinal analysis demonstrated a significant age and grade-dependent shift in astigmatism composition. As illustrated in Figure 1, the prevalence of mild, moderate, and severe astigmatism generally increased with age and grade, whereas the proportion of physiological astigmatism (< 0.75 D) showed a corresponding decline (Age: χ^2^ = 6,434.391, *P*_*trend*_ < 0.001; Grade: χ^2^ = 5,883.680, *P*_*trend*_ < 0.001).This developmental pattern was further characterized by the distribution of astigmatism severity across educational stages (Table 3 and Figure 2). A Pearson's chi-square test revealed significant differences in severity distribution among primary, middle, and high school students (χ^2^ = 5,212.356, *P* < 0.001). Pairwise comparisons highlighted divergent trajectories for different severity groups: specifically, the prevalence of mild astigmatism increased significantly from primary (27.1%) to junior high school (35.6%, *P* < 0.05) but plateaued during the transition to high school (34.5%, *P* > 0.05). In contrast, moderate and severe astigmatism exhibited a continuous and significant upward trend throughout all educational stages (all *P* < 0.05).

### Grade-dependent trends in astigmatism axis

**Figure 3:**
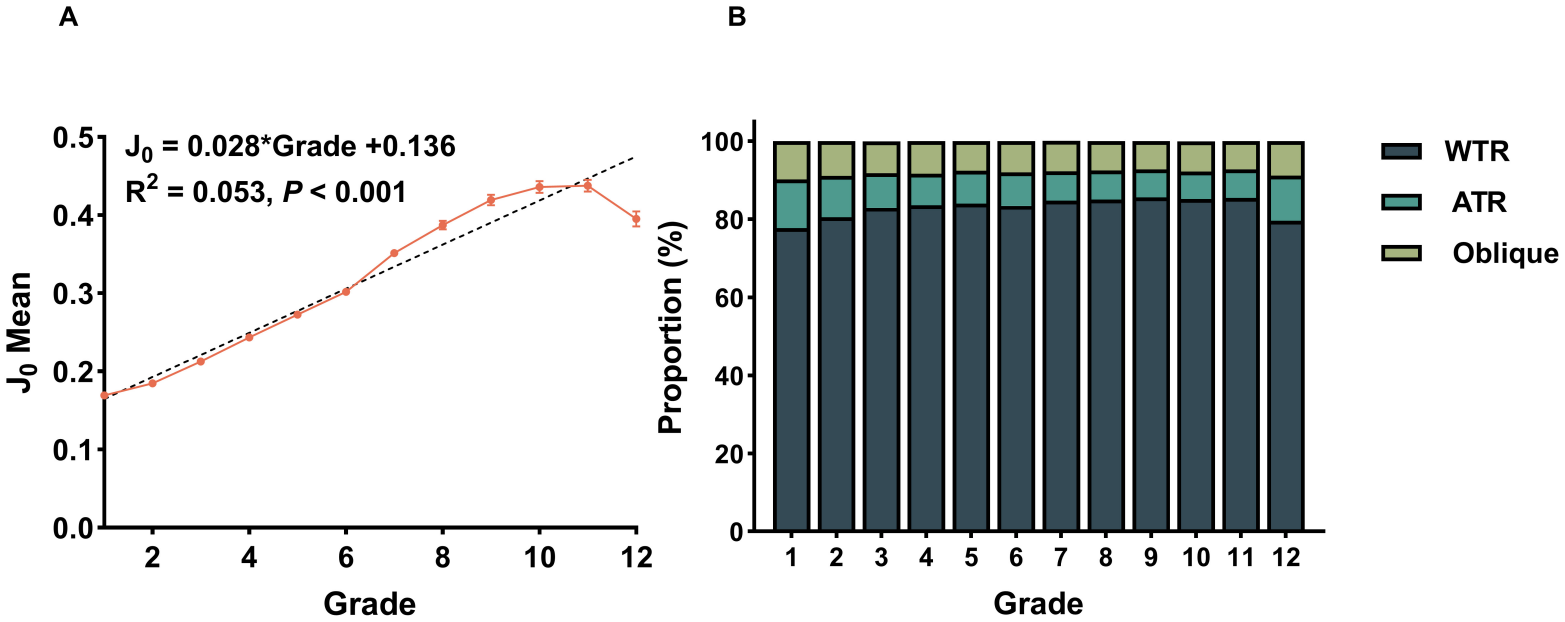
**(A)** Longitudinal trend of the astigmatic vector component J_0_ across different grades. The dashed line represents the linear regression fit, demonstrating a highly significant positive trend (*F* = 4,398.435, *P* < 0.001) of increasing WTR components with advancing grade levels. **(B)** Proportional shift in astigmatism axis distribution stratified by school grade. The stacked bar chart depicts the composition of astigmatism axes from Grade 1 to Grade 12 indicating a progressive shift toward WTR dominance as students advance in grade level.

Vector analysis of the astigmatic component *J*_0_demonstrated a significant positive linear correlation with grade level (*F* = 421.347, *P* < 0.001, $\eta_{p\;}^{2}=$ 0.055, indicating a moderate effect size), Figure 3A. This vector trend was paralleled by significant variations in the clinical distribution of axis types (χ^2^= 372.930, *P* < 0.001). As detailed in Table 4, WTR was the pre-dominant subtype throughout the entire study population (82.9%) and consistently maintained dominance across all individual grade levels. Specifically, trend analysis confirmed a robust linear increase in the prevalence of WTR astigmatism, rising from 77.7% in Grade 1 to 85.4% in Grade 11 ($\chi_{\text{trend}}^{2}$ = 104.436, *P* < 0.001, Figure 3B). Conversely, the proportions of ATR and Oblique astigmatism showed corresponding linear declines over the same period, with a slight fluctuation in these trends observed in Grade 12.

**Table 4:** Astigmatism axial distribution in different grades.

Grade	Astig_type [N (%)]			χ^2^	*P*-value	*P_*trend*_*
	WTR	ATR	Oblique			
1	6,641 (77.7)	1,066 (12.5)	840 (9.8)	372.930	< 0.001	< 0.001
2	8,246 (80.5)	1,084 (10.6)	911 (8.9)			
3	6,692 (82.9)	722 (8.9)	654 (8.1)			
4	6,239 (83.6)	608 (8.1)	618 (8.3)			
5	6,764 (84.0)	680 (8.4)	610 (7.6)			
6	5,733 (83.4)	593 (8.6)	549 (8.0)			
7	6,646 (84.8)	586 (7.5)	609 (7.8)			
8	5,191 (85.0)	459 (7.5)	456 (7.5)			
9	4,250 (85.6)	358 (7.2)	356 (7.2)			
10	3,367 (85.2)	278 (7.0)	305 (7.7)			
11	3,387 (85.4)	295 (7.4)	284 (7.2)			
12	2,379 (79.6)	348 (11.6)	262 (8.8)			
Total	65,535 (82.9)	7,077 (9.0)	6,454 (8.2)			

WTR, with-the-rule; ATR, against-the-rule.

### Independent determinants of astigmatism orientation

**Figure 4:**
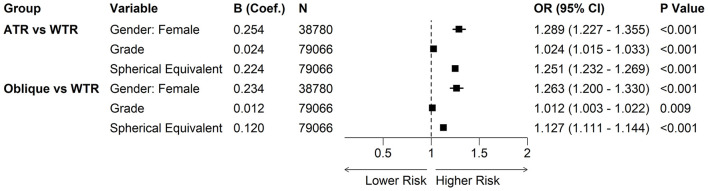
Multinomial logistic regression analysis of factors associated with astigmatism axis types. The forest plot displays the Odds Ratios (*OR*) and 95% Confidence Intervals (CI) for factors associated with ATR and Oblique astigmatism, using WTR astigmatism as the reference outcome. Squares: point estimates of the *OR*. Horizontal lines: 95% CI. Vertical dashed line: Represents an *OR* of 1 (no effect). Values to the right (>1) indicate a higher risk compared to the WTR group, while values to the left (< 1) indicate a lower risk. Predictors: analysis was adjusted for Gender (Reference: male), Grade, and Spherical Equivalent (*SE*).

Multinomial logistic regression was performed to identify factors associated with astigmatism axis types, using WTR as the reference group (Figure 4). The analysis revealed that gender, grade level, and *SE* were all independently associated with the axis orientation. Compared to males, females had significantly higher odds of presenting with ATR (*OR* = 1.289, 95% CI: 1.227–1.355, *P* < 0.001) and Oblique astigmatism (*OR* = 1.263, 95% CI: 1.200–1.330, *P* < 0.001). Furthermore, a more positive *SE* (indicating less myopia or more hyperopia) was associated with increased odds of both ATR (*OR* = 1.251, 95% CI: 1.232–1.269, *P* < 0.001) and Oblique (*OR* = 1.127, 95% CI: 1.111–1.144, *P* < 0.001) subtypes. After adjusting for *SE* and gender, advancing grade levels were independently associated with slightly increased odds of ATR (*OR* = 1.024, 95% CI: 1.015–1.033, *P* < 0.001) and Oblique astigmatism (*OR* = 1.012, 95% CI: 1.003–1.022, *P* < 0.009).

### Impact of astigmatism on visual acuity

Stratified multiple linear regression analyses were performed to evaluate the association between astigmatism magnitude and visual acuity across different levels of myopia, adjusting for spherical power and age. The unstandardized coefficients (*B*) for the non-myopic, low myopia, moderate myopia, and high myopia groups were 0.063 (95% CI: 0.059 to 0.067, standardized β = 0.242, *P* < 0.001), 0.102 (95% CI: 0.098 to 0.106, standardized β = 0.223, *P* < 0.001), −0.004 (95% CI: −0.010 to 0.002, standardized β = −0.013, *P* = 0.120), and −0.015 (95% CI: −0.023 to −0.007, standardized β = −0.060, *P* < 0.001), respectively.

**Figure 5:**
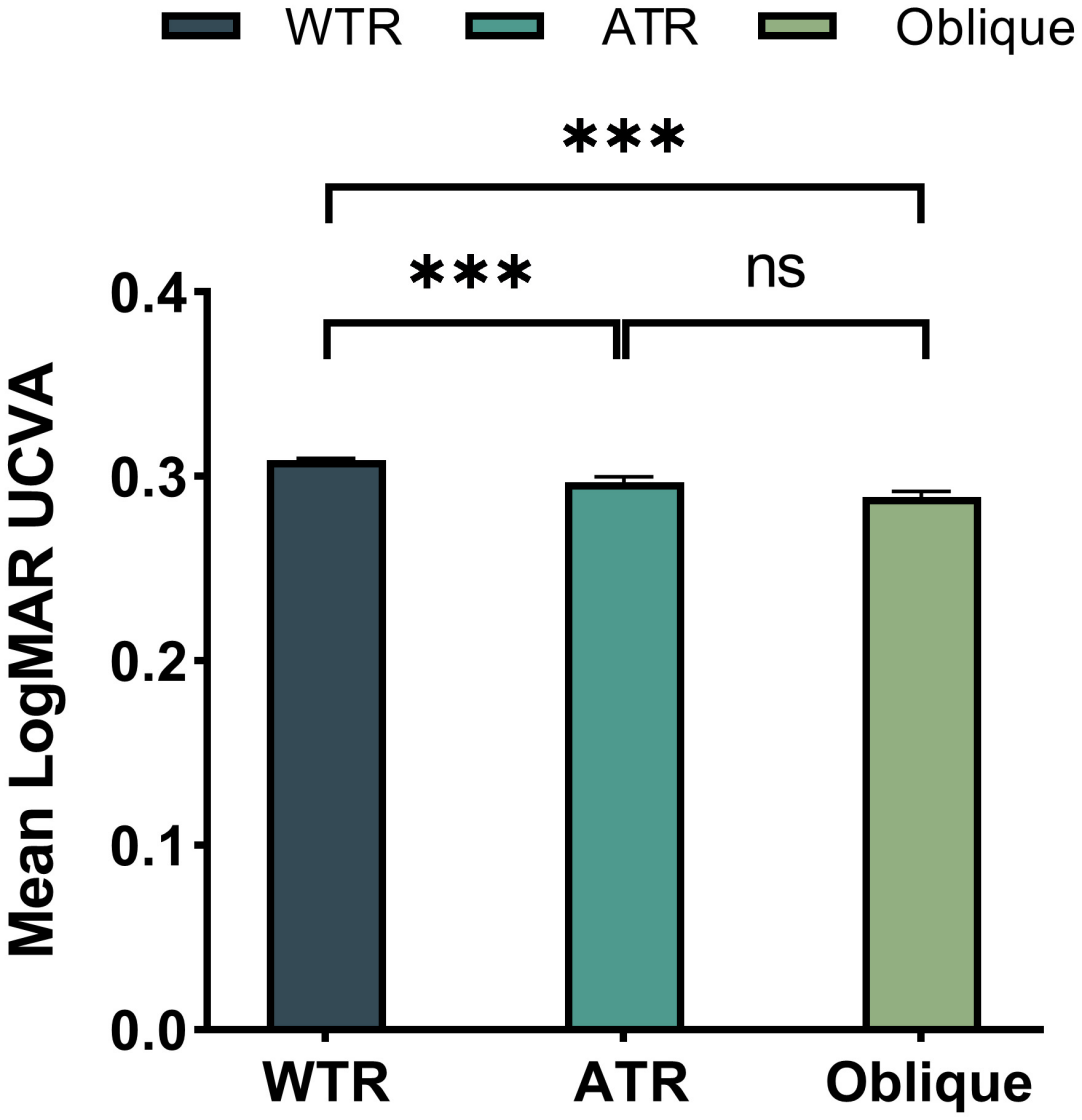
Impact of astigmatism axis on uncorrected visual acuity (UCVA). Error bars represent the 95% confidence interval (CI). ***P < 0.001, ns, no statistically significant difference was observed.

A GLM was employed to assess the influence of astigmatism axis on visual acuity, controlling for potential confounders including astigmatism magnitude, spherical power and age. The estimated marginal means for LogMAR visual acuity were 0.309 for WTR, 0.297 for ATR, and 0.289 for Oblique astigmatism. *Post-hoc* pairwise comparisons revealed that, with the exception of the comparison between ATR and Oblique (*P* = 0.118), all other differences were statistically significant (*P* < 0.001, Figure 5); however, the effect size of astigmatism axis on visual acuity was clinically negligible ($\eta_{p}^{2}$ = 0.001).

## Discussion

This large-scale cross-sectional study of 79,066 children and adolescents in Beijing revealed an overall astigmatism prevalence of 43.6%, pre-dominantly characterized by mild severity and the WTR subtype. Stratified analyses demonstrated that both the prevalence and severity of astigmatism increased progressively with advancing educational stages, accompanied by a distinct shift toward the WTR axis. Furthermore, sex, grade level, and *SE* emerged as significant independent factors associated with astigmatic orientation. Crucially, the impact of astigmatism magnitude on UCVA was found to be highly modulated by the concurrent level of myopia. After rigorous adjustment for confounders, WTR astigmatism was associated with a slight visual disadvantage. While this may partially reflect a specific interaction between meridional blur and optotype design, it is equally plausible that such a minuscule difference stems from the practical variations and statistical amplifications inherent in real-world, large-scale mass screenings. Together, these findings not only map the epidemiological trajectory of pediatric astigmatism but also highlight its complex interplay with the concurrent myopization process and visual acuity, thereby providing essential insights for targeted clinical management.

## Prevalence and geographic disparities

The overall prevalence of astigmatism in our cohort was 43.6%, which marks a dramatic increase compared to early data from Beijing (10%−15% in 2000) (11). Our findings align closely with recent post-pandemic data from Hong Kong (46.5%) (12) and mainland surveys in Zibo (46.21%) (10) and Xinjiang (13), though it remains lower than the 73.81% reported in Shaanxi (7). In contrast, Western populations consistently report substantially lower rates. For instance, a US study found a prevalence of 16.8% even among higher-risk Asian and Hispanic subgroups (14), and a recent European meta-analysis reported 16.29% (15). These marked discrepancies likely reflect a combination of genetic pre-dispositions, the intensifying educational burden unique to East Asia, and variations in diagnostic criteria (e.g., employing ≥ 1.00 D or ≥ 1.50 D thresholds in Western studies).

## Severity progression and the “ceiling effect”

Regarding severity, mild astigmatism constituted the majority of cases (30.1%), followed by moderate (11.2%) and severe (2.3%) forms, consistent with previous domestic reports (16). Overall astigmatism prevalence increased significantly with advancing educational stage, escalating from 35.1% in primary school to 55.8% in junior high, and reaching 60.9% in senior high school (5–7, 13, 16, 17) This age-dependent trajectory intimately mirrors the broader myopization process (10, 18, 19) and cumulative near-work stress extensively documented in East Asian populations (1, 20, 21).

Notably, a distinct divergence emerged during the transition to senior high school: while the prevalence of mild astigmatism plateaued, moderate and severe forms exhibited a continuous upward trend. This “ceiling effect” for mild cases likely reflects the physiological deceleration of ocular growth and emmetropization post-puberty (22). Conversely, the persistent rise in severe forms suggests that while the onset of new mild cases may decelerate, the progression of existing astigmatism continues unabated under sustained academic pressure (17). Clinically, these patterns define the primary and junior high school years (ages 6–14) as the critical window for preventing astigmatism onset. For senior high school students, clinical vigilance must instead shift toward monitoring and controlling the progression of existing cases to prevent visual impairment associated with high astigmatism (23).

## Astigmatism axis and the confounding role of myopia

Our findings confirm that WTR astigmatism remains the pre-dominant subtype (82.9%) across all educational grades, consistent with previous studies in Chinese children (5, 7, 24). Univariate analysis revealed a progressive ‘WTR shift' with advancing grades, accompanied by a decline in ATR and oblique components. Mechanistically, this crude trend is traditionally attributed to intensified near-work demands, where prolonged reading imposes vertical compressive forces on the cornea via increased eyelid tension (10, 25, 26). However, our multivariate regression analysis revealed a critical nuance: after adjusting for *SE*, advancing grade was independently associated with a slight shift toward ATR and oblique astigmatism. This apparent discrepancy between the crude and adjusted analyses highlights a classic confounding effect. Physiologically, the natural, age-dependent structural evolution of the eye—such as decreased upper eyelid tension or lenticular changes—inherently favors an ATR shift (27, 28). However, during the school-aged years, this subtle anatomical ATR progression is completely optically and biomechanically overwhelmed (masked) by the massive concurrent myopization process (axial elongation), which strongly drives a WTR shift (20). Consequently, the overwhelming WTR shift observed in the crude analysis is primarily a byproduct of myopia progression rather than chronological age itself. Nevertheless, the precise interactive patterns and underlying mechanisms between WTR astigmatism and myopization warrant further investigation.

## Sexual dimorphism in astigmatism

Regarding demographic factors, our gender-stratified analysis highlighted a distinct male susceptibility, with boys demonstrating a significantly elevated risk for moderate-to-severe astigmatism (7, 29). Furthermore, independent of refraction, multivariate analysis revealed that males possess a higher pre-disposition to WTR astigmatism, whereas females exhibit a greater probability of developing ATR and oblique forms. This sexual dimorphism may stem from inherent anatomical differences, such as tighter eyelid tension or narrower palpebral fissures in males (30, 31), which reinforce the mechanical molding of the vertical corneal meridian. Given that astigmatism—particularly the environmentally promoted WTR subtype—can hinder academic performance (32), these findings underscore the need for sex-specific strategies in clinical vision screening and longitudinal myopia management.

## Impact of astigmatism magnitude and axis on visual acuity

Our results demonstrate that the impact of astigmatism magnitude on UCVA is strongly modulated by the concurrent spherical refractive status. In non-myopic and low-myopic eyes, astigmatism emerged as a significant determinant of visual degradation (B=0.063 and 0.102, respectively). This suggests an ‘additive blur' effect, wherein astigmatic defocus exacerbates the visual deficit associated with early-stage myopia. These findings align with established optical models [1, 2], confirming that astigmatism disproportionately impairs resolution when spherical defocus is mild. Conversely, this effect diminished to non-significance in moderate myopia (P=0.120) and exhibited a slight reversal in high myopia. This corroborates the ‘blur saturation' hypothesis [3], suggesting that profound spherical defocus masks the distinct contribution of astigmatism. Clinically, these data imply that while spherical error dominates visual outcomes in high myopia, strict astigmatism correction is paramount in low myopia to prevent excessive visual loss.

Beyond magnitude, we evaluated the influence of the astigmatic axis on visual performance. After adjusting for astigmatism magnitude, spherical power, and age, UCVA in the WTR group was slightly worse than in the oblique group (P < 0.001). Despite this statistical significance, the absolute difference of 0.02 LogMAR is clinically negligible, representing less than one-fifth of a line on a standard visual acuity chart. Such a small effect size likely reached high statistical significance simply due to the massive sample size of our cohort. As previously established, visual performance across different astigmatic axes varies depending on the typographic features of the optotype and the orientation of meridional blur (33). For example, Kobashi et al. (9) found no significant difference in reading performance between WTR and ATR astigmatism using Japanese optotypes, while Serra et al. (33) reported that nearly 15% of subjects tested with Tamil script achieved better acuity with oblique astigmatism than with ATR.

In our study, the slight visual disadvantage in the WTR group likely reflects the specific optical properties of the Tumbling E chart (34); alternative optotypes, such as the Landolt C or complex Chinese characters, might yield different meridional sensitivities. WTR astigmatism primarily causes vertical image smear, which selectively degrades the horizontal gaps necessary for identifying the directional orientation of the “E” (33, 35). However, considering the real-world nature of this mega-cohort study, alternative explanations must be critically acknowledged. A mean difference of 0.02 LogMAR—representing less than one-fifth of a line on a visual acuity chart—could readily be influenced by minor environmental fluctuations during mass school screenings (e.g., ambient classroom lighting), transient fatigue among students, or simply represent a statistically amplified “noise” driven by the massive sample size. Ultimately, while the astigmatism axis exerts a detectable statistical effect on visual performance, the magnitude of astigmatism remains the more substantial determinant of UCVA in this population, even though its overall impact is secondary to that of spherical error (8).

The primary strength of this study lies in its large-scale, school-based design involving nearly 80,000 children, which provides massive statistical power to detect subtle trends in astigmatism evolution. Furthermore, the application of continuous vector analysis (J_0_ and J_45_ alongside conventional categorical notation allowed for a more rigorous quantification of astigmatic changes that might be obscured by simple magnitude analysis. Additionally, this study distinguishes itself from many previous cross-sectional surveys—which pre-dominantly focused on epidemiological prevalence and demographic risk factors (5, 7, 17)—by comprehensively evaluating the specific impact of the astigmatic axis on UCVA and robustly adjusting for the confounding effect of myopization.

However, several limitations must be acknowledged. First, refractive measurements were obtained using non-cycloplegic autorefraction, which introduces a potential measurement bias. While cycloplegia is the gold standard for assessing spherical power in children, previous studies consistently indicate that cylindrical power and axis are relatively stable and significantly less influenced by accommodation than spherical equivalents (36–38). Therefore, our findings regarding astigmatic patterns and meridional distribution remain highly valid. Second, potential selection bias should be noted. Although our sample size is massive, it was drawn exclusively from schools in Beijing. Consequently, these findings may not be completely generalizable to pediatric populations in rural areas or other geographic regions with different genetic backgrounds and educational burdens. Third, while our multivariate models rigorously adjusted for spherical power and age, residual confounding factors remain a limitation. Critical environmental and genetic variables—such as parental refractive history, daily outdoor activity time, and specific near-work duration—were not collected during this mass screening but could significantly influence astigmatic development. Finally, the cross-sectional design inherently restricts causal inference regarding individual progression. Although the observed grade-level trends strongly suggest a dynamic longitudinal shift, our research team is currently undertaking an in-depth longitudinal cohort study to specifically validate this ‘WTR shift' and elucidate its definitive causal relationship with myopia development and axial elongation.

## Conclusion

In conclusion, WTR is the pre-dominant astigmatism subtype among school-aged children, with its prevalence increasing alongside advancing educational stages and myopization. Males exhibit a higher susceptibility to overall astigmatism and the WTR orientation, whereas females are more prone to ATR and Oblique forms. Regarding visual performance, the magnitude of astigmatism is the primary determinant of UCVA degradation, heavily modulated by the concurrent spherical refractive status, whereas the impact of the astigmatic axis is clinically insignificant. Consequently, our cross-sectional findings suggest that targeted astigmatism monitoring and correction strategies may benefit from being tailored to specific genders and refractive profiles within comprehensive pediatric myopia management programs.

## Data Availability

The raw data supporting the conclusions of this article will be made available by the authors, without undue reservation.
